# An alteration in the expression of cell wall structural proteins increases cell surface exposure of adhesins to promote virulence in *Candida glabrata*

**DOI:** 10.1128/msphere.00910-24

**Published:** 2024-11-27

**Authors:** Yaling Zhang, Shengwei Gong, Kang Xiong, Xiangtai Yu, Xinreng Mo, Chang Su, Yang Lu

**Affiliations:** 1 Hubei Key Laboratory of Cell Homeostasis, College of Life Sciences, TaiKang Center for Life and Medical Sciences, Wuhan University, Wuhan, China; 2 Hubei Key Laboratory of Cell Homeostasis, College of Life Sciences, Wuhan University, Wuhan, China; Shenzhen Institute of Synthetic Biology, Chinese Academy of Sciences, Shenzhen, China

**Keywords:** *Candida glabrata*, cell wall remodeling, adhesins, virulence

## Abstract

*Candida glabrata* is an opportunistic human fungal pathogen that causes superficial mucosal and life-threatening bloodstream infections in immunocompromised individuals. Remodeling in cell wall components has been extensively exploited by fungal pathogens to adapt to host-derived stresses, as well as immune evasion. How this process contributes to *C. glabrata* pathogenicity is less understood. Here, we applied RNA sequencing and an *in vivo* invasive infection model to elucidate the prompt response of *C. glabrata* during infection. Fungal transcriptomes show a dramatic alteration in the expression of Srp1/Tip1-family cell wall structural proteins during systemic infection. Deletion of all six genes in this family (*TIR2-5* and *AWP6-7*) that are upregulated during infection leads to a significantly lower fungal burden in organs, as well as an attenuated virulence in the dextran sulfate sodium-induced colitis model. The *tir2-5 awp6-7* sextuple mutant does not display any defect in response to host-derived stresses. Rather, deletion of all these six genes results in a lower cell surface exposure of an adhesin Epa1, which could contribute to its reduced adhesion to epithelial cells and cytotoxicity, as well as attenuated virulence. Our study reveals that cell wall remodeling triggered by the alteration in the expression of structural proteins is a key virulence attribute in *C. glabrata* that facilitates this fungus adhering to host cells and persisting in organs.

**IMPORTANCE:**

*Candida glabrata* is one of the most frequent causes of candidiasis after *Candida albicans*. While *C. albicans* has been extensively studied, the mechanisms of infection and invasion of *C. glabrata* have not been fully elucidated. Using an infection model of systemic candidiasis and RNA sequencing, we show that there is a dramatic change in the expression of Srp1/Tip1-family genes during infection. Deletion of all six Srp1/Tip1-family genes that are upregulated during infection decreases the amount of cell wall-localized Epa1, probably reflecting the reduced adherence to epithelial cells and attenuated virulence in the sextuple mutant. These data suggest that alterations in the expression of Srp1/Tip1-family structural proteins trigger cell wall remodeling that increases the cell surface exposure of adhesins, such as Epa1, to promote virulence. Our study provides a pathogenic mechanism associated with *C. glabrata* in ensuring its sustenance and survival during infection.

## OBSERVATION

*Candida glabrata* (*Nakaseomyces glabrata*), a ubiquitous commensal of humans, primarily colonizes the oral cavity and gastrointestinal tract in most healthy individuals (1). On the other hand, this fungus emerges as an important pathogenic yeast, capable of causing superficial to life-threatening systemic infections with high morbidity and mortality rates. Despite the importance, it is still unclear how this fungus responds to the host environment to successfully establish the invasion during the systemic infection.

**Fig 1:**
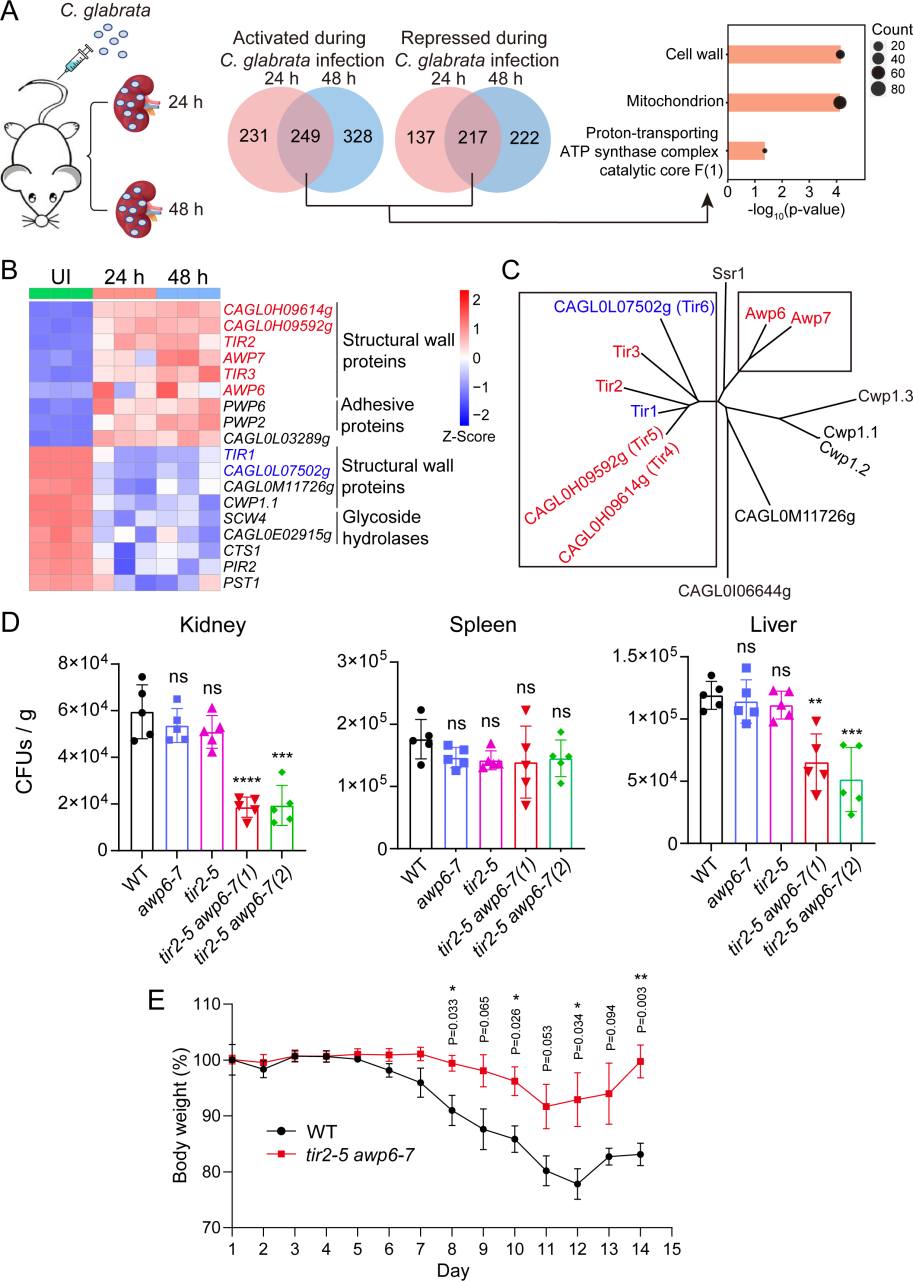
Deletion of all six Srp1/Tip1-family genes that are upregulated during infection results in attenuated virulence. (**A**) Venn diagram of gene expression showing the overlap of gene sets with significant expression changes based on the RNA-Seq analysis for *C. glabrata* genes at 24 h and 48 h post-invasive infection. Activation was defined by a minimum twofold increase of gene expression at 24 h pi or 48 h pi and repression by a minimum twofold decrease. The false discovery rate was filtered to 0.05. Kyoto Encyclopedia of Genes and Genomes (KEGG) component enrichment analysis of the overlapping genes is shown on the right. (**B**) Heatmap showing the expression levels of genes for cell wall in panel **A**, in *z*-score normalized to fragments per kilobase per million mapped reads. UI, uninfected. (**C**) Phylogenetic tree of the predicted structural wall proteins in *C. glabrata*. Fourteen *C. glabrata* glycosylphosphatidy-1-inositol (GPI) proteins have been found to have the homology to cell wall proteins of *Saccharomyces cerevisiae* that are suggested to have a structural function. (**B** and **C**) Srp1/Tip1-family genes highlighted in red indicate upregulation during infection, while those in blue indicate downregulation. (**D**) Groups of immunosuppressed male BALB/c mice were infected with 5 × 10^7^ colony forming units (CFU) of wild type, *tir2-5* quadruple mutant, *awp6-7* double mutant, or *tir2-5 awp6-7* sextuple mutant by tail vein injection, followed by euthanasia of five animals per group after 4 days. Two independent *tir2-5 awp6-7* sextuple mutant strains were used for the experiment. BALB/c mice were rendered neutropenic by intraperitoneal administration of cyclophosphamide (150 mg/kg of body weight per day) 3 days before the challenge and on the day of infection. CFUs were determined by plating kidney, liver, or spleen homogenates onto agar plates (supplemented with streptomycin and ampicillin) and counting after incubation at 30°C. The data are represented as mean ± standard deviation (SD). (**E**) The body weight loss in mice colonized with wild type *C. glabrata* or the *tir2-5 awp6-7* sextuple mutant strain during the induction of colitis by low doses of dextran sulfate sodium (DSS). Female c57 mice (8–10 weeks old) were infected by oral gavage with 5 × 10^7^ cells of wild type or *tir2-5 awp6-7* sextuple mutant, and treated with 1.5% DSS on the day of infection. The DSS administration occurred over a period of 2 weeks. The values represent means *±* SEM for *n* = 4 mice per group. (D and E) Significance was measured with the two-tailed unpaired *t*-test in GraphPad Prism. ns, nonsignificant; *P < 0.05, **P < 0.01, ***P < 0.001, ****P < 0.0001.

To investigate the prompt response of *C. glabrata* during the invasive infection, RNA-Seq analysis was applied in a murine model (Fig. 1A), where *C. glabrata* is injected intravenously and spreads to several tissues. The number of viable organisms in the kidney was determined at 24 h post-infection (pi) and 48 h pi (Fig. S1A). Periodic acid–Schiff staining showed that fungal cells existed as yeast form in histological sections at both time points (Fig. S1B). However, leukocytes were not recruited to the loci where *C. glabrata* cells were accumulated (Fig. S1B). Principal component analysis on the transcriptome profiles confirmed the general differences between infected (24 h and 48 h) and uninfected (UI) samples (Fig. S1C). Using a twofold cutoff, there are 480 genes upregulated and 354 genes downregulated upon invasive infection for 24 h, and the numbers are 577 and 439 at 48 h pi (Fig. 1A). We then focused on 249 upregulated genes and 217 downregulated genes which are co-regulated at 24 h pi and 48 h pi and subjected them to KEGG analysis. As shown in Fig. 1A, the sets were significantly enriched in genes encoding components for cell wall (*P* = 7.01 × 10^−5^), mitochondrion (*P* = 8.75 × 10^−5^), and proton-transporting ATP synthase complex catalytic core *F*(1) (*P* = 0.0446).

Cell wall proteins, including adhesins, invasins and hydrolases, are key virulence attributes in pathogens, among which adhesins serve as pivotal virulence factors in *C. glabrata* (2). In contrast to our expectation, only two adhesins, Pwp2 and Pwp6, exhibited an elevated expression during the invasive infection (Fig. 1B). Interestingly, the expression of all members of the Srp1/Tip1-family cell wall structural proteins identified to-date is changed at both 24 h pi and 48 h pi. It is reported that eight cell wall proteins in *C. glabrata* harbor the domain architecture for *S. cerevisiae* members of Srp1 (serine-rich protein)/Tip1 (temperature-inducible protein) family, which are suggested to have a structural function (3–5). Besides the domain architecture for Srp1/Tip1 family, all Srp1/Tip1 proteins feature a signal peptide at the N-terminus and a C-terminal GPI-anchor (Fig. S2A). Among them, *TIR2* (*CAGL0F01485g*), *TIR3* (*CAGL0C03872g*), *CAGL0H09614g*, *CAGL0H09592g*, *AWP6* (*CAGL0G10175g*), and *AWP7* (*CAGL0C00209g*) were upregulated, whereas *TIR1* (*CAGL0F01463g*) and *CAGL0L07502* were downregulated (Fig. 1B). We next performed the phylogenetic analysis for the predicted structural wall proteins in *C. glabrata* with MEGA11 software (Fig. 1C). According to this set of comparisons, six of the Srp1/Tip1 family proteins, *TIR1*, *TIR2*, *TIR3*, *CAGL0H09614g*, *CAGL0H09592g*, and *CAGL0L07502*, cluster together phylogenetically, and they are designated as TIP1-related (TIR) cluster. The genes *CAGL0H09614g*, *CAGL0H09592g*, and *CAGL0L07502* in this cluster are named as *TIR4*, *TIR5*, and *TIR6*, respectively. Two other genes *AWP6* and *AWP7* display higher homology and are upregulated during infection. Notably, the clustering of homology does not correspond to the expression pattern during the invasive infection of *C. glabrata: TIR1* and *TIR6* are downregulated, while others in the TIR cluster are upregulated (Fig. 1C). Hence, our transcription profiling data indicate an alteration in the expression of cell wall structural components during invasive infection of *C. glabrata*.

Real-time quantitative reverse transcription PCR (qRT-PCR) analysis at 24 h pi and 48 h pi confirmed the expected change in the expression of Srp1/Tip1-family genes during infection, as well as under the tissue culture condition (Fig. S2B). Because *TIR4* and *TIR5* differ by only five nucleotides in sequence, we used the same primers to amplify both *TIR4* and *TIR5*. Considering several Srp1/Tip1-family genes in *S. cerevisiae* are upregulated in response to cold shock and anaerobiosis (6), we asked whether it is the case in *C. glabrata*. Unexpectedly, the expression of *TIR3*, *TIR4*, *TIR5*, *AWP6*, and *AWP7* was relatively low upon a downshift in growth temperature from 30°C to 13°C compared to that in kidneys, while the *TIR2* and *TIR6* transcripts increased under the same condition (Fig. S2B). Although we detected an increase in the expression of *TIR2*, *TIR3*, *TIR4*, *TIR5*, *AWP6*, and *AWP7* under the anaerobic condition, their induction is not as dramatic as that during *in vivo* infection (Fig. S2B).

To elucidate the role of Srp1/Tip1 family in *C. glabrata*, each gene in this family was deleted individually. Since *TIR4* and *TIR5* share very high homology, we designed a single guide RNA (sgRNA) that targets both genes. The six single mutants and a *tir4-5* double mutant displayed no obvious growth defect in response to the cell-wall-perturbing agents, including SDS, Congo red and KCl (Fig. S3A). We then focused on the Srp1/Tip1-family genes whose expression increased during the invasive infection. Considering evolutionary relationship among Srp1/Tip1-family members, we constructed the *tir2-5* quadruple mutant strain, *awp6-7* double mutant strain, and the sextuple mutant (*tir2-5 awp6-7*) in which all Srp1/Tip1-family genes that are upregulated during infection (*TIR2-5* and *AWP6-7*) were deleted. The *tir2-5*, *awp6-7*, and *tir2-5 awp6-7* mutant strains grew normally on media containing cell wall stress agents (Fig. S3A). Similarly, no defect was observed in these mutant strains when grown under anaerobic condition (Fig. S3B). These data indicate that these genes do not impact *C. glabrata* adaptation to cell wall stress and hypoxia.

We next compared two independent *tir2-5 awp6-7* sextuple mutants to WT, *tir2-5* or *awp6-7* strain in tissue fungal burdens, which were measured in the immunosuppressed mouse model using tail-vein injections according to the previously established protocol (7). As shown in Fig. 1D, both sextuple mutants exhibited significant lower fungal loads compared to the other three strains in kidneys and livers, but not in spleens. Thus, Srp1/Tip1 family is critical for *C. glabrata* to defend against clearance from organs during infection. We then investigated the impact of Srp1/Tip1-family genes on virulence in the intestinal tract. Since all reported *C. glabrata* strains are eliminated within 2 days after challenge from mice without colitis, the dextran sulfate sodium (DSS)-induced colitis model was used to experimentally mimic chronic inflammatory bowel diseases. Notably, DSS-treated mice inoculated with the WT *C. glabrata* experienced a greater loss of body weight starting from day 8 compared to those inoculated with the *tir2-5 awp6-7* sextuple mutant strain (Fig. 1E). This result suggests that WT *C. glabrata* causes more severe colitis than the sextuple mutant, although weight loss is an indirect readout of the colitis model.

**Fig 2:**
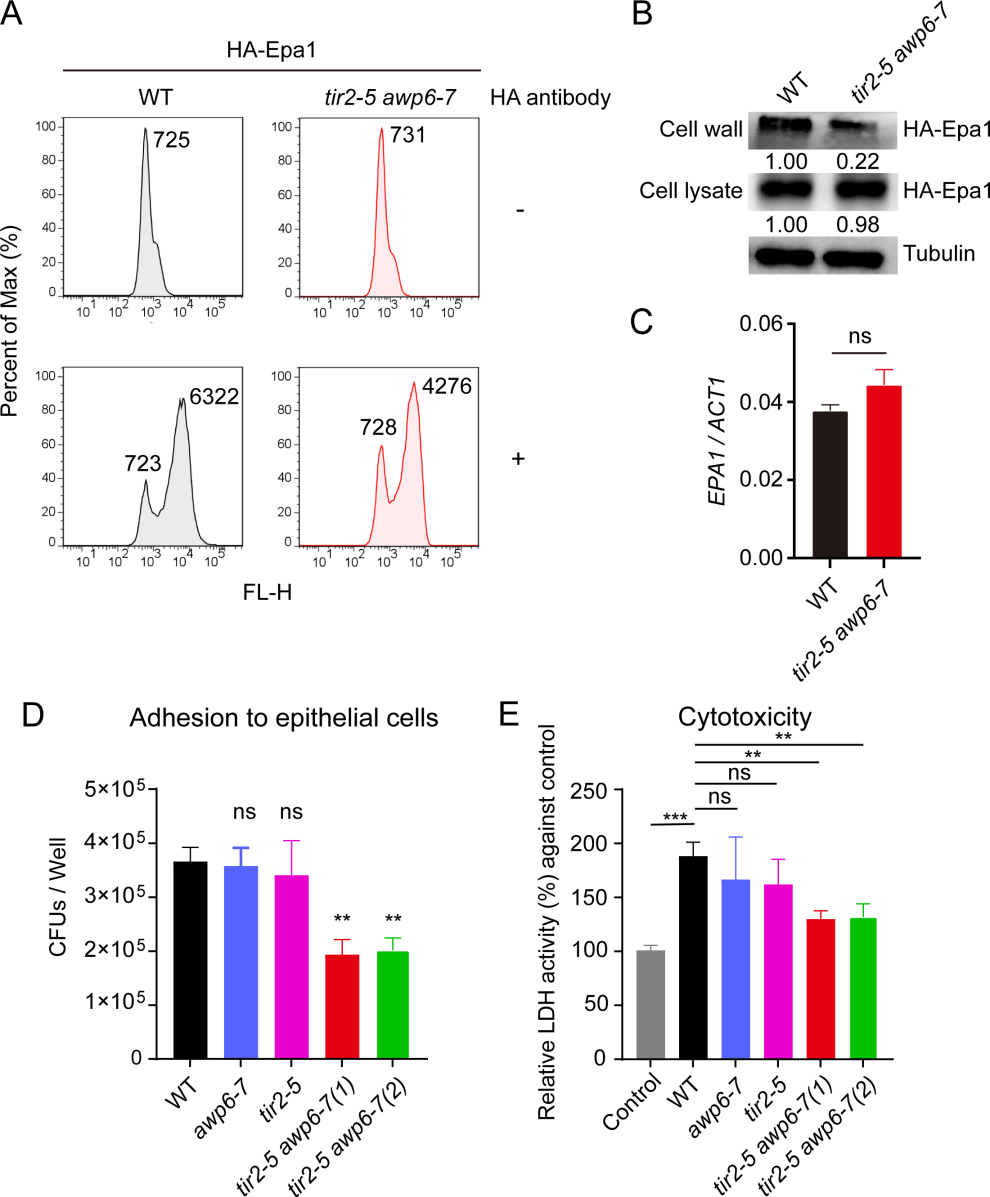
The reduced cell surface exposure of Epa1 in the *tir2-5 awp6-7* sextuple mutant. (**A**) Fluorescence-activated cell sorting (FACS) analysis of HA-Epa1 fusion proteins on the cell surface in WT strain (black) and *tir2-5 awp6-7* sextuple mutant (red). *C. glabrata* cells carrying HA-Epa1 from the *TEF1* promoter were incubated in Dulbecco's modified Eagle medium (DMEM) supplemented with 10% serum at 37°C in 5% CO_2_. Surface exposure was monitored by immunofluorescence after labeling with anti-HA antibody and fluoresceine isothiocyanate (FITC)-conjugated secondary goat anti-mouse antibody. No primary antibody control was included. Average fluorescence of the population was indicated. Plots are representative of data collected in two independent replicate experiments. (**B**) Western analysis of HA-Epa1 fusion protein in the cell wall fraction of WT and *tir2-5 awp6-7* sextuple mutant cells incubated in DMEM medium with 10% serum at 37°C in 5% CO_2_. Band intensities were quantified and shown as the ratio of indicated protein vs tubulin. Representative plots of three independent experiments are shown. (**C**) Real-time quantitative reverse transcription PCR (qRT-PCR) analysis for *EPA1* expression in *C. glabrata* cultured as described in (**B**). The expression levels of *EPA1* were showed as means ± SD. *n* = 3 biologically independent experiments. (**D**) Adhesion assay on epithelial cell monolayers. *C. glabrata* cells of wild type and indicated mutant strains were inoculated with A549 cells in DMEM medium supplemented with 10% serum. After incubation for 4 h, non-adherent *Candida* cells were removed by washing with phosphate-buffered saline (PBS). The numbers of adherent *Candida* cells are represented as means ± SD from three independent experiments. (**E**) Cytotoxicity assays. A549 cell damage following treatment with wild type, *tir2-5* quadruple mutant, *awp6-7* double mutant, or *tir2-5 awp6-7* sextuple mutant was determined after co-incubation for 24 h. Relative LDH activity (%) against A549 cells without *C. glabrata* cells was calculated. Mean data ± SD from three independent experiments was plotted. (**C–E**) Significance was determined using the two-tailed unpaired Student’s *t*-test. ns, nonsignificant. **P < 0.01, ***P < 0.001.

How Srp1/Tip1 family impacts *C. glabrata* virulence? We hypothesize that alterations in the expression of Srp1/Tip1-family structural proteins might trigger cell wall remodeling that increases cell surface exposure of adhesins as the capacity of adhesion to host cells represents a major virulence attribute of *C. glabrata*. Exploiting the fact that *C. glabrata* adhesion to host cells largely depends on the GPI-anchor protein Epa1 (8, 9), we measured the localization of hemagglutinin (HA)-tagged Epa1 in WT and *tir2-5 awp6-7* sextuple mutant by surface antibody labeling, and median fluorescence intensity was quantified by flow cytometry. As shown in Fig. 2A, a reduction in cell surface exposure of Epa1 in the *tir2-5 awp6-7* sextuple mutant was observed compared to that in WT strain. Supporting this, Western analysis indicated a less amount of Epa1 from the cell wall in *tir2-5 awp6-7* sextuple mutant than in WT strain (Fig. 2B). In contrast, deletion of the Srp1/Tip1-family cell wall structural proteins did not impact β-glucan exposure (Fig. S4) and displayed comparable *EPA1* expression in both transcription and protein levels to WT cells (Fig. 2C). The adherence assay on the human epithelial cells A549 revealed that *tir2-5 awp6-7* sextuple mutant exhibited reduced attachment compared to the wild type (Fig. 2D). Consistently, *tir2-5 awp6-7* sextuple mutant strain induced less damage to A549 cells than WT *C. glabrata* as indicated by the lower lactate dehydrogenase (LDH) activity (Fig. 2E). Thus, Srp1/Tip1-family members are critical for cell wall localization of GPI-linked adhesin, Epa1, which facilitates *C. glabrata* adhering to host cells and developing invasion.

*C. glabrata* has a large repertoire of cell wall-associated adhesins (10–12). Yapsin (YPS)-family aspartyl proteases are implicated in *C. glabrata* cell wall remodeling by the removal of GPI-linked adhesin Epa1 (13). Unexpectedly, we did not detect a significant change in the expression of adhesins and YPS-family aspartyl proteases during infection. Instead, a significant change in the expression of all eight members in Srp1/Tip1-family cell wall structural proteins was observed during *C. glabrata* infection. Deletion of Srp1/Tip1-family genes that are upregulated during infection resulted in a reduction in Epa1 adhesin at the cell wall periphery but did not affect *EPA1* expression. We suggest that dysfunction in the regulation of cell wall structural proteins might result in aberrant surface localization of adhesins, which could contribute to the attenuated virulence. The mechanism of how cell wall structural proteins regulate cell surface localization of adhesins awaits further investigation. In addition to Epa1, activation of Srp1/Tip1 genes might affect other aspects implicated in pathogenicity. Regardless of the mechanism, our study reveals a critical role of cell wall structural proteins in *C. glabrata* virulence.

### Media and growth conditions

*C. glabrata* was routinely cultured in yeast extract peptone dextrose (YPD) medium (2% Bacto peptone, 2% glucose, 1% yeast extract) at 30°C. Transformants were selected on YPD plates supplemented with 200 µg/mL nourseothricin or synthetic medium (0.17% Difco yeast nitrogen base without ammonium sulfate, 0.5% ammonium sulfate, and auxotrophic supplements) with 2% glucose. The anaerobic condition was generated using an anaerobic rectangular jar and AnaeroPack-Anaero (Mitsubishi Gas Chemical Company, Inc.).

To determine the stress response of *C. glabrata*, freshly grown cells were serially diluted 10-fold, spotted onto YPD plates with or without 1 mg/mL Congo red, 1.5 M KCl, or 0.05% SDS and incubated for 1–3 days at 30°C.

### Plasmid and strain construction

CBS138 genomic DNA was used as the template for all PCR amplifications of *C. glabrata* DNA. *C. glabrata* strains used in this study are listed in Table S1. The primers used for PCR amplification are listed in Table S2.

The CRISPR-Cas9 strategy was used for the deletion of *C. glabrata* genes as follows. The sgRNA was annealed to insert into the BsmBI site of the vector pV1382 (14). The resulting plasmid was transformed into *C. glabrata* cells with the repair template. The mutants were verified by sequencing.

*C. glabrata URA3* was deleted using SAT1-flipping strategy (15). Upstream (primers 5 and 6) and downstream (primers 7 and 8) sequences of *URA3* were cloned as ApaI-XhoI and NotI-SacI fragments, respectively, on both sides of the *SAT1* flipper cassette to obtain the plasmid pSFS2-URA3 for *URA3* disruption. The pSFS2-URA3 plasmid was linearized using ApaI and SacI, and transformed into *C. glabrata* cells to disrupt the endogenous copy of *URA3*.

The DNA sequence encoding the signal peptide of Epa1 and the HA tag was synthesized by GENEWIZ and inserted into the XbaI-BamHI site of plasmid pYC121 (16) to create pYC121-SP-HA. Subsequently, primers 3 and 4 were used to amplify the coding sequence for Epa1 without the signal peptide from *C. glabrata* genomic DNA. The PCR product was inserted into the BamHI-SalI site of pYC121-SP-HA, generating pYC121-HA-EPA1 plasmid to express HA-Epa1 fusion protein under the control of *TEF1p* in *C. glabrata*.

### Adhesion of *C. glabrata* to human epithelial cells

Adhesion assays were performed by following a modified protocol described previously (17) and by using the epithelial human cell line A549. A549 epithelial human cells were cultured in DMEM medium supplemented with 10% fetal bovine serum (FBS) at 37°C in 5% CO_2_. For the adhesion assay, *C. glabrata* cells, suspended in DMEM medium supplemented with 10% FBS (1 × 10^6^ cells), were added to the 12-well plate containing monolayers of A549 cells and cultured at 37°C in 5% CO_2_. After incubation for 4 h, non-adherent *C. glabrata* cells were carefully removed by washing with PBS. Epithelial cells and adherent *C. glabrata* cells were visualized by Giemsa staining.

### Damage assay

The release of LDH into the culture supernatant was monitored as a measure of the damage to epithelial cells. Experiments were performed in 24-well plates at 37°C in 5% CO_2_. For control samples, epithelial cells were incubated with medium only. After co-incubation with *C. glabrata* for 24 h, culture supernatants were collected, and the amount of LDH was determined using the Cytotoxicity Assay (Promega G1780) according to the manufacturer’s instructions. The absorbance of the blank medium was subtracted as the background value from the observed absorbance of each sample. The relative activity was plotted as a percentage of the control samples.

### Murine model of systemic infection

BALB/c and c57 mice were purchased from Beijing Vital River Laboratory Animal Technology Company. Mice were housed in a temperature-constant animal room (22°C) with a reversed dark/light cycle (7:00 a.m. on and 7:00 p.m. off) and 40%–70% humidity.

Male BALB/c mice (6–8 weeks old) were rendered neutropenic by intraperitoneal administration of cyclophosphamide (150 mg/kg of body weight per day) 3 days before challenge with *C. glabrata* infection and on the day of infection. *C. glabrata* cells were injected into immunosuppressed mice via tail vein injection at a dose of 5 × 10^7^. On day 4 post-infection, the infected kidneys, spleens, and livers were dissected, and the tissue homogenates were plated on agar plates containing streptomycin (100 µg/mL) and ampicillin (50 µg/mL). Fungal colony forming units were counted after incubation at 30°C.

### Induction of DSS colitis

The DSS-induced colitis experiment was performed in 8–10-week-old female C57BL/6J mice as described previously with modifications (18, 19). Mice were administered 1.5% DSS (36–50 kDa; MP Biomedicals) in drinking water in order to induce intestinal inflammation. The DSS water was used throughout the DSS-induced colitis experiment for 2 weeks (from day 1 to day 14). On day 1, mice were inoculated with 5 × 10^7^ *C. glabrata* cells by oral gavage. The body weight of the mice was measured on a daily basis, and the data are presented as the mean percentage change from the initial body weight.

### RNA sequencing and analysis

For RNA-seq assay during *C. glabrata* infection, 6–8-week-old male BALB/c mice were inoculated with 4.5 × 10^8^ wild-type cells (CBS138) in a 200-µL volume of sterile PBS via tail vein injection. At 24 h and 48 h post-infection, animals were euthanized. The infected kidneys from two mice were randomly picked from a certain group and combined as a sample for RNA extraction, with UI *C. glabrata* cells incubated in the YPD medium at 30°C as the control. DNA-depleted RNA samples were then depleted of ribosomal RNA using the Ribo-Zero rRNA Removal Kit (Epicentre) according to the manufacturer’s protocol. Sequencing was performed using the Illumina nova6000 platform. Clean reads were selected from raw reads by removing reads with adapter and low quality. Q30 and GC content of clean data were calculated. The sequencing reads were then aligned to *C. glabrata* reference genome (http://www.candidagenome.org/) using HISAT2 with default parameters, and the aligned reads were assembled and quantified. Genes with zero counts were excluded. Differentially expressed genes were defined by fold change ≥2 and a false discovery rate <0.05 found by DESeq2. KEGG analysis was performed on the DAVID online platform (20, 21).

### FACS assay and Western analysis

Surface Epa1 was detected by FACS and Western blot as described in the published study (9). The WT and *tir2-5 awp6-7* sextuple mutant strains carrying HA-Epa1 were incubated in DMEM medium supplemented with 10% serum at 37°C in a 5% CO_2_ incubator. For FACS analysis, these cells were collected and labeled with mouse anti-HA antibody (MBL) for 30 min at 4°C on a rotating wheel. FITC-conjugated goat anti-mouse antibody (ABclonal) was used as the secondary antibody for the immunofluorescence labeling assay. The negative control without primary antibody (mouse anti-HA antibody) was included. *C. glabrata* cells were then washed three times in PBS and resuspended in 250 µL of PBS for FACS analysis using a CytoFLEX (Beckman).

The cell wall fraction was prepared for the detection of Epa1 localization by Western blot. Cells of each sample were pelleted and resuspended in 0.45 mL of lysis buffer (50 mM Tris-HCl, pH 7.5, 150 mM NaCl, 0.1% NP40, 1 mM PMSF) plus protease inhibitor cocktail (Roche). Cells were lysed with acid-washed glass beads (Sigma-Aldrich) using FastPrep (MP Biomedicals) at a setting of 5.0 with four pulses of 3 min intervals. The supernatant was obtained for the analysis of tubulin expression as a loading control using β-tubulin antibody (Proteintech). To collect cell wall and membrane material, broken cells were washed from the glass beads, and the extract was pelleted at 15,000 × *g* for 10 min. The pellet was then boiled for 10 min in 50 mM Tris-HCl buffer containing 2% SDS (pH 7.5) to remove the SDS-extractable material. The cell debris was washed with ice-cold water. Cell wall proteins were then released from the washed cell wall material by treatment with Endo-1,3-β-glucanase (TargetMoI) and probed for HA-Epa1 using anti-HA antibody (MBL).

### Quantitative PCR analysis

Total RNA was extracted and purified from *C. glabrata* cells incubated under the *in vitro* conditions using the RNeasy Mini Kit. To remove DNA contamination, the RNA samples were treated with RNase-free DNase Set (Qiagen) for 15 min at room temperature. To determine the expression of *C. glabrata* genes during invasive infection, 6–8-week-old male BABL/c mice were inoculated with 4.5 × 10^8^ live *C. glabrata* cells by tail vein injection, and infected kidneys were taken at 24 h after injection. Total RNA in the infected kidneys of mice was extracted using the RNAprep Pure Tissue Kit (Tiangen). cDNA was synthesized using the Maxima H Minus cDNA Synthesis Master Mix with dsDNase (Thermo). For qPCR analysis, iQ SYBR Green Supermix (Bio-Rad) was used in 96-well plates. The primers for qRT-PCR are listed in Table S2. Signals obtained from *ACT1* mRNA were used for normalization. All data are shown as the mean of three independent experiments with error bars representing the standard deviation (SD).

### Staining and quantification of exposed β-glucan

The WT and *tir2-5 awp6-7* sextuple mutant strains were grown in DMEM medium supplemented with 10% serum at 37°C in a 5% CO_2_ incubator. Staining of exposed β-1,3-glucan in *C. glabrata* cells was performed using a primary dectin-Fc antibody (MedChemExpress) and a secondary anti-IgG Fc antibody conjugated to FITC (BioLegend) as described previously (22). The secondary antibody-only control was included. Cells were then fixed in 4% formaldehyde (methanol-free), diluted in FACS buffer, and analyzed using a flow cytometer. Ten thousand events were acquired for each sample. Average fluorescence intensity is indicated for each sample as determined using FlowJo software.

### Statistical and reproducibility

All experiments were performed with at least three biological repeats, and no statistical method was used to predetermine sample sizes. No data were excluded from analyses. Analyses were conducted using Graphpad Prism. Results are expressed as the mean ± SD except as indicated in the figure legends. All data were analyzed using unpaired Student’s *t*-test. *P* values of less than 0.05 were considered statistically significant. Sample allocation was random in all experiments.

## Data Availability

RNA-Seq data that support the findings of this study have been deposited into GEO under the accession code GSE279281.

## SUPPLEMENTAL MATERIAL

Supplemental material msphere.00910-24-s0001.pdf Fig. S1-S4; Tables S1 and S2.

ASM does not own the copyrights to Supplemental Material that may be linked to, or accessed through, an article. The authors have granted ASM a non-exclusive, world-wide license to publish the Supplemental Material files. Please contact the corresponding author directly for reuse.
