# Supplementary material for: An alteration in the expression of cell wall structural proteins increases cell surface exposure of adhesins to promote virulence in Candida glabrata
Source: mSphere. 2024 Nov 27;9(12):e00910-24. doi: 10.1128/msphere.00910-24 (PMC11656777; doi:10.1128/msphere.00910-24)
Supplement: Supplemental material — Fig. S1-S4; Tables S1 and S2. [file msphere.00910-24-s0001.pdf]

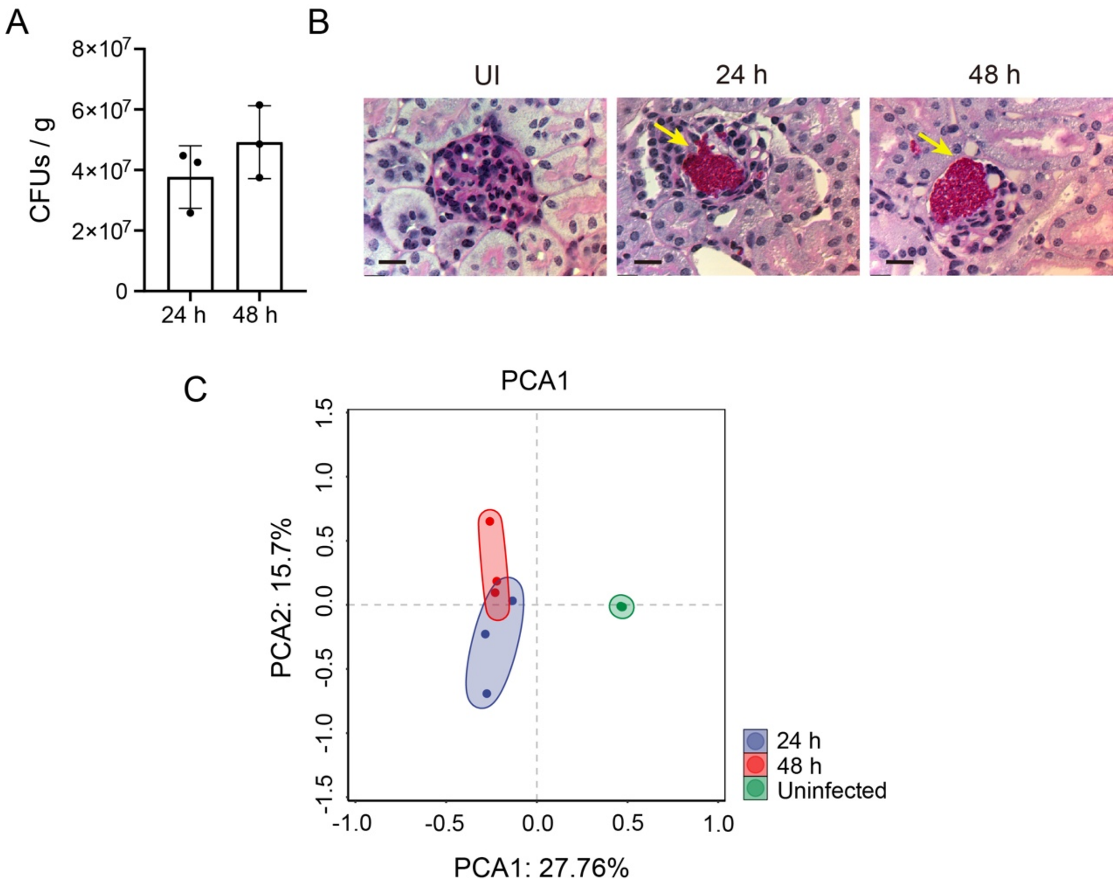

FIG S1 *In vivo* RNA sequencing. (A) Fungal burdens of WT *C. glabrata* (CBS138) at 24 h and 48 h pi for RNA-seq analysis. (B) WT *C. glabrata* grew in yeast form in host kidneys. The images of PAS-stained kidneys from mice infected with WT *C. glabrata* via tail vein injection. Yellow arrows indicate yeast-form *C. glabrata* cells. Scale bar, 20 μm. UI, uninfected. (C) Principal-component analysis (PCA) of WT *C. glabrata* under in vitro condition (Uninfected, *n* = 3 cultures), and in murine kidneys at 24 h and 48 h post infection (*n* = 3 samples at each time point).

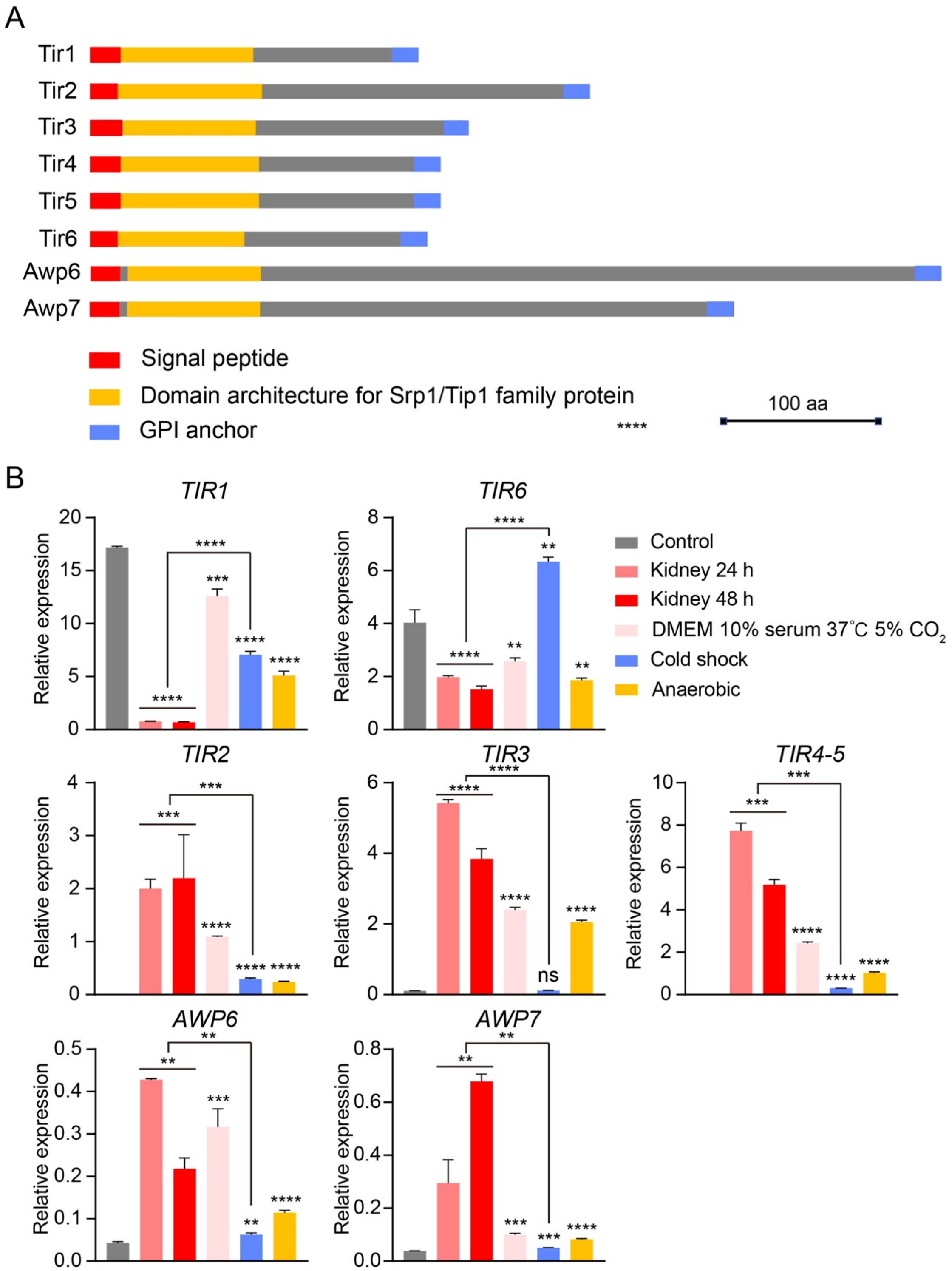

FIG S2 Expression patterns of the Srp1/Tip1 family genes under *in vivo* and *in vitro* conditions. (A) Schematic depiction of signal peptide (red), the domain architecture for *S. cerevisiae* members of Srp1/Tip1 family (yellow), and GPI anchor (blue) in *C. glabrata* Srp1/Tip1 family proteins. aa, amino acids. (B) qRT-PCR analysis for the expression of Srp1/Tip1 family genes under different conditions. WT *C. glabrata* was grown in YPD

medium at 30°C for an in vitro control. At indicated times post infection, total RNA of the infected kidneys was extracted for the in vivo analysis. WT *C. glabrata* cells were subjected to the indicated treatment, including tissue culture condition (DMEM, 37°C, 10% serum, 5% CO_2), cold shock (13°C) and anaerobiosis, to analyze the expression of Srp1/Tip1 family genes. The signals obtained from *ACT1* mRNA were used for normalization. Error bars represent standard deviations from the means of three independent experiments. Significance was measured with the two-tailed unpaired *t* test in GraphPad Prism; ns, nonsignificant; **, $p < 0.01$; ***, $p < 0.001$; ****, $p < 0.0001$.

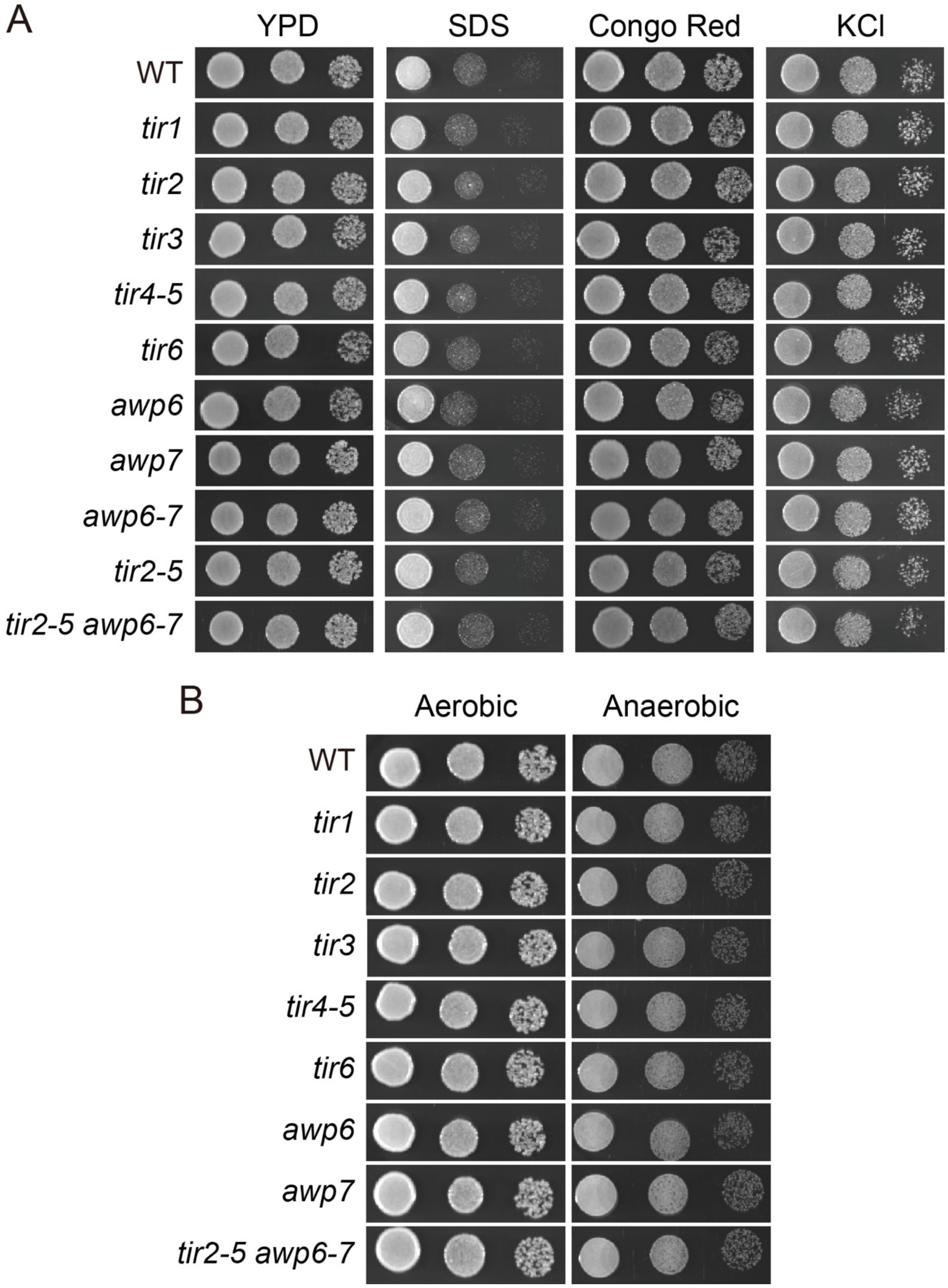

FIG S3 Srp1/Tip1 family members are not implicated in responses to cell wall stress agents and hypoxia. (A) The Srp1/Tip1 family genes are not required for cell wall

stress tolerance. Wild type and indicated mutant strains were treated with cell wall stress agents, including 1 mg/ml Congo red, 1.5 M KCl, and 0.05% SDS, and incubated at 30°C. (B) Cells of wild type, single mutants *tir1*, *tir2*, *tir3*, *tir6*, *awp6*, and *awp7*, double mutant *tir4-5*, and sextuple mutant *tir2-5 awp6-7* were serially diluted 10-fold and spotted onto YPD solid medium. The plates were incubated under anaerobic or normal air condition. Photographs were taken after 24 h of growth at 30°C. The growth assays were performed with at least three biological repeats.

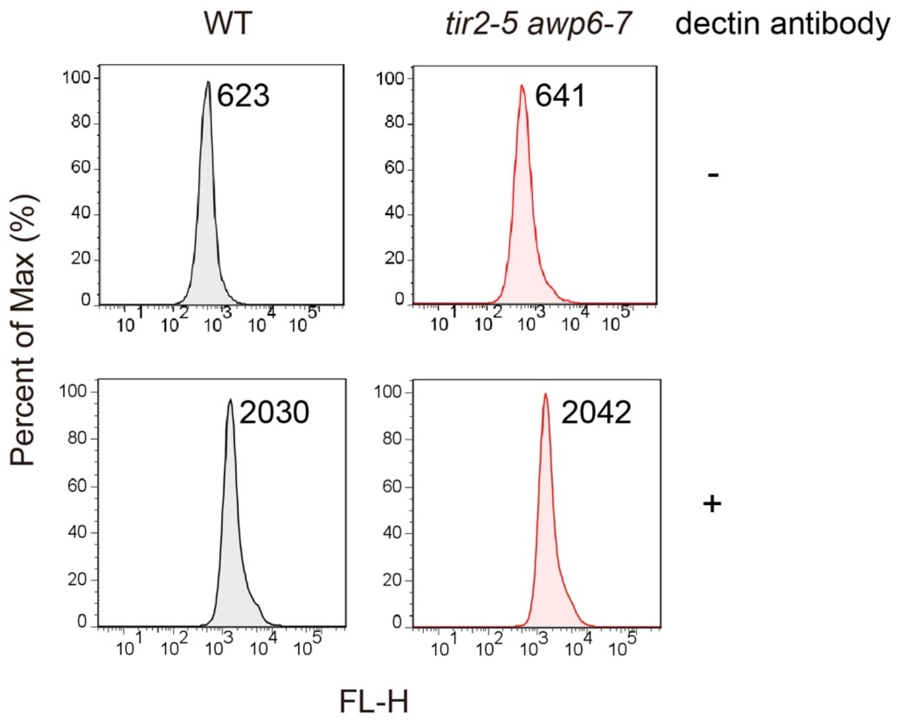

FIG S4 Flow cytometry analysis of β-glucan exposure in WT (black) and *tir2-5 awp6-7* sextuple mutant (red) grown in DMEM medium supplemented with 10% serum at 37°C in 5% CO_2. No primary antibody control was included in the experiment. The average fluorescence intensity was indicated. Plots are representative of data collected in two independent replicate experiments.

TABLE S1 *C. glabrata* strains used in this study

Strains	Genotype	Source
CBS138	Wild type	ATCC collection
YLC98	*tir1Δ*	This study
YLC99	*tir2Δ*	This study
YLC100	*tir3Δ*	This study
YLC101	*tir4Δ tir5Δ*	This study
YLC102	*tir6Δ*	This study
YLC103	*awp6Δ*	This study
YLC104	*awp7Δ*	This study
YLC107	*tir2Δ tir3Δ tir4Δ tir5Δ awp6Δ awp7Δ*	This study
YLC108	*awp6Δ awp7Δ*	This study
YLC109	*tir2Δ tir3Δ tir4Δ tir5Δ*	This study
YLC111	*ura3Δ::SAT1*	This study
YLC112	*tir2Δ tir3Δ tir4Δ tir5Δ awp6Δ awp7Δ ura3Δ::SAT1*	This study

TABLE S2 Primers used in this study

	Sequence (5'-3')	Purpose and features
1	TGCTCTAGAATGATTTTAAATCCAGCTCTATTTTTG	pYC121-HA-EPA1
2	CGCGGATCCAGCGTAATCTGGAACATCGTATGGGTAACCAGCGTAATCTGGAACATCGTATGGGTAACCAGCGTAATCTGGAACATCGTATGGGTATGTAGCATAACCACCATTCGTTAATAG	
3	ACGATGTTCCAGATTACGCTTCTTCCAATGATATCAGTTT	
4	TTCACCGTTAATTAACCCTATTAGGTCCCTATGTTCATC	
5	CTAGGGCCCAAGTGTCGTTAAGATCAATC	*URA3*-NAT-UF
6	GTACTCGAGTGTAAGTGTACTCTTGATGGT	*URA3*-NAT-UR
7	ATAAGAATGCGGCCGCTTGACACTTTGGGTCCATAC	*URA3*-NAT-DF
8	CGAGCTCCCTTCGTGCACATTTGGTTCC	*URA3*-NAT-DR
9	GATCGAGTGGGAGGAGAAGTCCTGTG	*TIR1* sgRNA
10	AAAACACAGGACTTCTCCTCCCACTC	
11	TTTCGCCGTTGCTCAAGACGCTACTCAATTGGCTGAATTGAACGAATTCTAATAAGACT	*TIR1* Repair template
12	TGGCCAACAAGCCGGTGTAGTCGGACAAGTGGGAGGAGAAGTCTTATTAGAATTCGTTC	
13	GATCGCAATTCAGCCAATTGCATTGG	*TIR2* sgRNA
14	AAAACCAATGCAATTGGCTGAATTGC	
15	CTGTCGCTCCTCTAGCTTTCGCTGCCGCTGTCGCTCATGCCTAATAACGAATTCCAATG	*TIR2* Repair template
16	CTTCAAGTCTTCCATGACGGCGTTCAATTCAGCCAATTGCATTGGAATTCGTTATTAGG	
17	GATCGCCATGTATAGATCCAAAACGG	*TIR3* sgRNA
18	AAAACCGTTTTGGATCTATACATGGC	
19	TTACATTTCCTACGCTGAAAACGATCCAAACTTCACCCTATAATAACTCGAGTTGGATC	*TIR3* Repair template
20	TGTAGGAGTCGTCAGTGGCGGTGGTCATAGCCATGTATAGATCCAACTCGAGTTATTAT	
21	GATCGGTCAATGTCCTTGTACAAGGG	*TIR4-5* sgRNA
22	AAAACCCTTGTACAAGGACATTGACC	
23	TTTGAACTTGGGTATGGCTTTGGCTTCCGCCACTGACGACTAATAAGAATTCTTGTACA	*TIR4-TIR5* Repair template

24	TGGACAAGAATGGAACAATACCATCGAAGTCAATGTCCTTGTACAAGAATTCTTATTAG	
25	GATCGACCTGCGTCAGCCAATCTTGG	*AWP6* sgRNA
26	AAAACCAAGATTGGCTGACGCAGGTC	
27	TGATTACAGGTGTCCCTTGGTATAGTGAAAGACTTGTTGGTGCTATTGAATTCTGATAG	*AWP6* Repair template
28	AGATGGAGATGGCCAGGCAGTAACAATACCTGCGTCAGCCTATCAGAATTCAATAGCAC	
29	GATCGTCAATCTCTCACTATACCAAG	*AWP7* sgRNA
30	AAAACTTGGTATAGTGAGAGATTGAC	
31	TGTCAGAGATTGCACCAGAAACTATCACAATGATGATTACAGGTGTG AAT TCG TAATAA	*AWP7* Repair template
32	ATCAGCAAGTCTACTAGCAATAGCACCGATCAATCTCTCTTATTACGAATTCACACCTG	
33	GATCGAGCGTCACCAGAACCAGCAGG	*TIR6* sgRNA
34	AAAACCTGCTGGTTCTGGTGACGCTC	
35	GTACTCCTCCAGATTGTTGCCAGCTCTAGAATCTGCCTCCTAAGAATTCGCTGGTTCTG	*TIR6* Repair template
36	AGGAAGCGTTAGCGGAAGCTGGGGCAGCGGCAGCGTCACCAGAACCAGCGAATTCTTAG	
37	CCGCTTTGGCTGCTTTCGC	*TIR1* qPCR
38	AGACCCAATTGTAGGACACCG	
39	TCCAATTGGGTATGGCCGTC	*TIR2* qPCR
40	CAGCGGCATATAGGGAGTCG	
41	CTTCACCCTACCAGACCACG	*TIR3* qPCR
42	GAATGGGGAGACGTTTGGCA	
43	ACCCCACAACAATTGGACGA	*TIR4 / TIR5* qPCR
44	AGCCAAAGCCATACCCAAGT	
45	CATCGCCTGTTCTCCCATCA	*AWP6* qPCR
46	TTGAGACTGTGGTGTTGCCA	
47	ATTGGCGCTGTCATGTTTGG	*AWP7* qPCR
48	CCATGCCGTTGCAATACCTT	
49	CGCTGCTATTGCTGCTAC	*TIR6* qPCR

50	GTCGTCAGTGTAAGTGGC	
51	ATGGATTCTGAAGTTGCTGC	*ACT1* qPCR
52	TGATACCTTGGTGTCTTGGT	
53	GATTATCATACAGAGACTCC	*EPA1* qPCR
54	CACAGATGAAGTAGATGATG	
